# Analysis of risk communication teaching in psychosocial and other medical departments

**DOI:** 10.1080/10872981.2020.1746014

**Published:** 2020-04-04

**Authors:** Franziska Baessler, Ali Zafar, Anja Ciprianidis, Fabienne Louise Wagner, Sonja Bettina Klein, Sophie Schweizer, Marina Bartolovic, Daniela Roesch-Ely, Beate Ditzen, Christoph Nikendei, Jobst-Hendrik Schultz

**Affiliations:** a Department of General Internal and Psychosomatic Medicine, Heidelberg University Hospital, Heidelberg, Germany; b Department of Gynecology and Obstetrics, Heidelberg University Hospital, Heidelberg, Germany; c Department of General Adult Psychiatry, Centre for Psychosocial Medicine, University of Heidelberg, Heidelberg, Germany

**Keywords:** Risk communication, medical education, medical curriculum, informed consent, communication skills, biostatistics

## Abstract

**Aims**: Teaching students about risk communication is an important aspect at medical schools given the growing importance of informed consent in healthcare. This observational study analyzes the quality of teaching content on risk communication and biostatistics at a medical school.

**Methods**: Based on the concept of curriculum mapping, purpose-designed questionnaires were used via participant observers to record the frequency, characteristics and context of risk communication employed by lecturers during teaching sessions for one semester. The data was analyzed quantitatively and descriptively.

**Results**: Teaching about risk communication was observed in 24.4% (n = 95 of 390) sessions. Prevalence varied significantly among different departments with dermatology having the highest rate (67.9%) but lesser in-depth teaching than medical psychology where risk communication concepts were discussed on a higher scale in 61.4% sessions. Relevant statistical values were not mentioned at all in 69% of these 95 sessions and clinical contexts were used rarely (55.8%). Supplementary teaching material was provided in 50.5% sessions while students asked questions in 18.9% sessions.

**Conclusions**: Students are infrequently taught about communicating risks. When they are, the teaching does not include the mention of core biostatistics values nor does the teaching involve methods for demonstrating risk communication.

## Introduction

### Background

Medical professionals are often required to interpret and communicate complex information about risks and benefits of diagnostics and therapies since medications and medical treatments carry inherent risks and side-effects. Similarly, patients and families need to understand these risks to give informed consent, discuss their concerns and plan for treatment [1]. The World Health Organization recognizes risk communication as communications and engagement with affected populations for taking informed decisions [2]. In the healthcare industry, risk communication is understood as ‘ … the open two-way exchange of information and opinion about harms and benefits, with the aim of improving the understanding of risk and of promoting better decisions about clinical management’ [3]. Risk communication is thus a core aspect of physician-patient relationship and a fundamental prerequisite for shared decision-making and therapy adherence [4–8].

Many physicians are however unable to communicate risks to patients effectively because of a lack of understanding of statistics as well as inadequate management of conflicts of interest [1,9–11]. Statistical illiteracy of medical professionals as a major cause of misinterpreting the harms and benefits of medical interventions [1,5,11–15] has been linked with a lack of statistics courses at medical schools and limited didactical expertise of teachers [16–18]. One study suggests that teaching basic statistical terms to medical students can not only help doctors better understand risk communication but also explain healthcare data and procedures to patients [19]. Although medical schools have incorporated risk communication teaching in curricula and also adopted innovative teaching approaches for improved knowledge transfer [20–22], literature on the quality of risk communication teaching and how students are taught is limited.

In Germany, most medical lawsuits which cite miscommunication as the primary liability cause are related to orthopedics and surgery procedures (42%) and internal and general medicine treatments (12%) [23]. This may explain the need and subsequent higher prevalence of risk communication content in surgery and internal medicine departments [24–26]. However, teaching risk communication is relevant for all medical departments and more research is needed outside the ‘major’ disciplines of medical schools.

### Aims and objectives

This study assesses the prevalence and depth of teaching content on risk communication in a medical curriculum. It focuses on the ‘smaller’ departments apart from surgery and internal medicine to observe whether medical students outside the larger specializations are taught about communicating risks and their relevant statistical values. The results of this extensive survey of medical courses could help identify knowledge gaps and practical implications. This could also help medical educators to design better teaching protocols and inclusive communicative methodologies and to develop future policies on appropriate risk communication teaching.

## Methods

### Study framework

This study was conducted at Heidelberg University Medical School during Winter Semester 2016/17, when 2,848 medical students were enrolled [27]. The Heidelberg University Medical School was ranked among the top universities in teaching communication skills to students [28]. The curriculum is comprised of 330 hours of communication courses in its six-year degree program with substantial content in Medi-KIT (Communication- and Interactive-Training Program), referred to as Simulated Patient-Training (SP-Training) hereafter [28,29].

Based on the premise of curriculum mapping, this study was based on five major observations:

1. Frequency of teaching content on risk communication;
2. Risk communication topics chosen by lecturers to teach students;
3. Depth of teaching content on communicating risks and biostatistics;
4. Mean time spent on discussing risk communication topics; and
5. Use of demonstration material to aid knowledge transfer.

A combined analysis of all these variables resulted in an extensive overview of the content and quality of risk communication teaching at a medical school.

### Study design

All teaching sessions (n = 390) at 14 departments were observed on a day-to-day basis. The surveyed departments were Epidemiology (including Epidemiology, Biostatistics and Informatics), Dermatology, Gynecology, General Medicine, Human Genetics, Health Economics, Medical Psychology, Neurology, Pediatrics, Radiology, Social Medicine and Psychiatry, Psychosomatic Medicine and Child and Adolescent Psychiatry (the last three offer combined classes).

### Observers

Twenty-seven (male = 12, female = 15) medical students, aged between 23 and 28 years, enrolled in the same degree program were recruited as participant observers. They were briefed on the study design and methodology, and invited for discussions to clarify any questions. The observers completed a questionnaire based on their observations, understanding and interpretation of the teaching content and methods employed by the lecturers to teach risk communication.

### Questionnaire

A study-specific questionnaire (RiskComm43 – Appendix) was designed based on the guides of Association for Medical Education in Europe (37, 43) through a literature review and interviews with medical educators. The German national medical education guidelines (National Competency-based Learning Objectives Catalogue in Medicine or NKLM) were used as a reference for identifying physician competencies and learning goals for medical students on risk communication. Six learning outcomes were identified through NKLM clauses and formulated into checklist items:

- ‘Diagnostic and therapeutic measures’ (NKLM 14 c.4.2.1);
- ‘Expected success, benefits, risks and costs’ (14 c.4.2.1)
- ‘Positive and negative consequences’ (14 c.2.6.2);
- ‘Uncertainty and evaluation models’ (14 c.4.2.3);
- ‘Sources of misjudgment’ (11.3.3.1); and
- ‘Characteristic factors of diagnostic tests’ (15.1.1).

A draft questionnaire was circulated among a team of 15 experts of medical education, communications and biostatistics and finalized through Delphi rounds (44). The final questionnaire included 19 descriptive and statistical values related to risk communication teaching, including specificity, sensitivity, positive predictive value, confidence interval, odds ratio, hazard ratio, survival/mortality rates, etc. and the descriptive values outlined above. The prevalence of these 19 values during a teaching session was rated on a scale similar to Miller’s Pyramid [30] beginning from the lowest a) not mentioned to the higher scales b) mentioned, c) reflected upon, d) transferred to a clinical context, and the highest e) set in practical context. The ratings ‘c’ and above were considered as intensive/in-depth teaching. The questionnaire was filled out by student observers whenever the lecturer used these characteristic statistical and descriptive concepts.

### Data processing and analysis

The observed teaching sessions (n = 390) consisted of 187 lectures, 121 seminars, 23 Practical Trainings, three Simulated Patient-Trainings (SP-Training), 21 Bed-Side Teachings (BST), 33 Problem-Based Learning (PBL) and two Skills Lab sessions. The observers submitted 278 questionnaires. Two questionnaires from elective courses were excluded since the teaching sessions were not part of the regular curriculum. For 112 sessions, the observers did not return questionnaires when they could not observe any risk communication teaching. For two teaching sessions, the observers submitted separate questionnaires for three discussion topics. For four teaching sessions, the observers submitted separate questionnaires for two discussion topics. These questionnaires were combined for data analysis.

Duplicate questionnaires were received for 68 sessions. Their inter-rater reliability was calculated at 71.64% and only one of the duplicate questionnaires was chosen randomly for analysis. Questionnaires with incorrect or missing factual information about teaching session or format were cross-checked with departmental timetables. Questionnaires for sessions without teaching content on risk communication were excluded from analysis.

The topics of risk communication used by lecturers were descriptively analyzed by two independent medical education experts through a coding system developed via consensus. The topics were placed within five thematic categories of a) diseases; b) prevention; c) treatment; d) consultation; and e) theoretical statistics. Overlapping themes were double-coded i.e., if a topic overlapped with another, such as ‘colon cancer screening’, it was coded for both categories of ‘diseases’ and ‘prevention’. For simplification of the broadest and highest prevailing topics of ‘disease’, it was divided into four sub-categories: a.1) cancer; a.2) brain; a.3) pregnancy; and a.4) others.

Microsoft Excel (v2010) was used to analyze statistical data. Open-text fields (comments section) were analyzed descriptively.

## Results

### Prevalence

**Figure 1.**
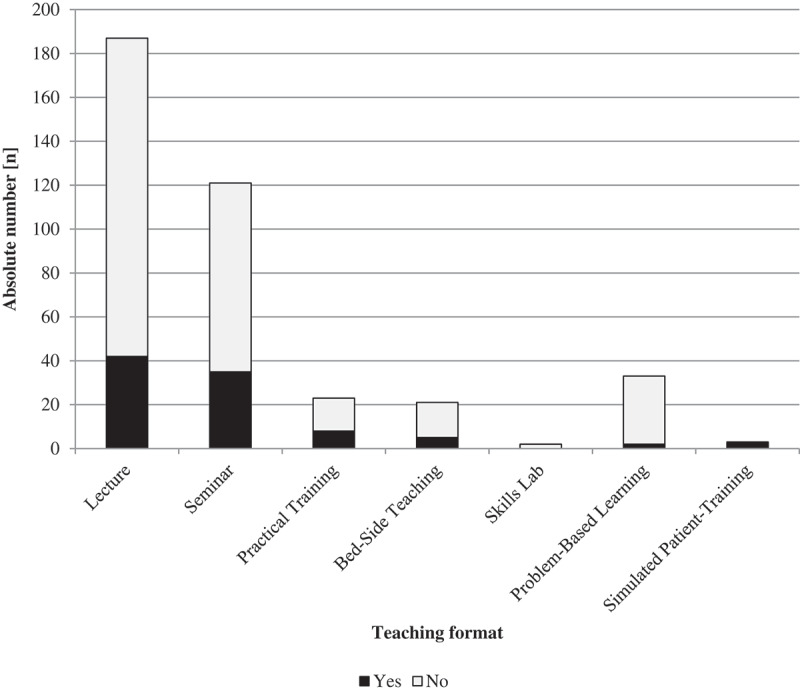
Prevalence of teaching content on risk communication in all teaching formats

Lecturers taught students about communicating risks in 24.4% (n = 95) of the 390 teaching sessions surveyed (Figure 1). In lectures, the prevalence of risk communication topics was 22.5% and 28.9% in seminars. Risk communication topics were at least mentioned in 34.8% of Practical Training sessions, 23.8% of BST sessions and 6.1% of PBL sessions. Risk communication topics were used in all three Simulated Patient-Training sessions. The prevalence was 0% in Skills Lab.

**Figure 2.**
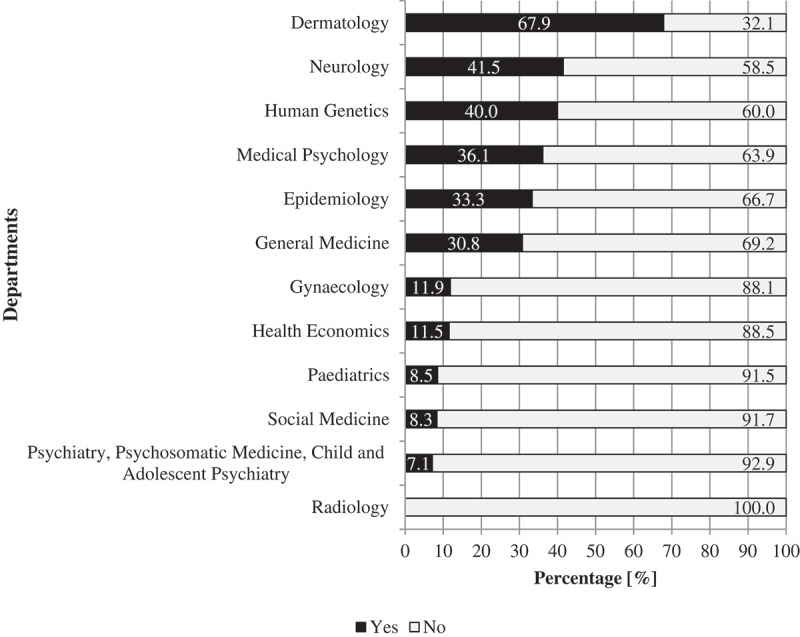
Frequency of risk communication teaching at every department

The Dermatology department had the highest prevalence of risk communication topics with 67.9%, followed by Neurology (41.5%) and Human Genetics (40%). In Medical Physiology, the prevalence was 36.1%. In Psychiatry, Psychosomatic Medicine and Child and Adolescent Psychiatry, it was 7.1%. Overall six out of 14 departments had a prevalence percentage above 30%. The results are summarized in Figure 2.

### Teaching topics

Risk communication was taught using 87 different topics. For 43 sessions, the observers did not provide this information. Lecturers used three different topics to teach risk communication within one teaching session two times and used two topics in six sessions. The topic category of ‘diseases’ was used 39 times; ‘prevention’ 15 times; ‘treatment’ 10 times; ‘consultation’ 14 times; and ‘theoretical statistics’ nine times. In the ‘prevention’ category, three topics were related to primary prevention with the majority accounting for secondary prevention. Under the ‘diseases’ category, the subcategory ‘relation to pregnancy’ was used 12 times; ‘cancer’ 12; ‘brain’ 9; and ‘others’ six times. The remaining subcategory included ‘flu’, ‘cardiovascular diseases’, ‘gastro-intestinal disorders’ and ‘diabetes mellitus’. Under the ‘cancer’ subcategory, mostly gynecological entities were used to teach risk communication while colon cancer and skin cancer were used thrice and once, respectively.

### Depth of teaching

The 19 statistical/descriptive categories used to measure the depth of teaching content on risk communication were at least ‘mentioned’ on average in 31.4% of the 95 teaching sessions and not mentioned in the remaining 69%. These values were ‘mentioned’ in 17%, ‘reflected upon’ in 5.8%, ‘transferred to a clinical context’ in 4.6% and ‘set in practical context’ on average in 4% of the observed teaching sessions.

Categories of ‘consequences’, ‘expected success’ and ‘diagnostic measures’ were taught above the ‘mentioned’ scale in more than 10% of the 95 sessions. In two-thirds of the sessions, lecturers did not ‘mention’ any of the following statistical values: ‘numbers needed to treat’, ‘uncertainty’, ‘absolute numbers’, ‘relative risks’, ‘sensitivity’, ‘survival/mortality rates’, ‘hazard ratio’, ‘odds ratio’, ‘confidence interval’, ‘p-value’, ‘positive predictive value’, ‘specificity’, ‘sources of misjudgment’, ‘consequences’ or ‘expected success’.

Teaching quality varied throughout the departments. In Dermatology, which had the highest prevalence of risk communication content, in 78.7% sessions lecturers did not mention any of the 19 statistical/descriptive values related to risk communication. It was taught on the lowest teaching level ‘just mentioned’ in 16.5%, ‘reflected upon’ in 2.1% and ‘transferred to a clinical context’ or ‘set in practical context’ in 5.5% of the sessions.

In Medical Psychology, risk communication was taught with none of the values in 22.8% sessions. It was taught on the lowest teaching level ‘mentioned’ in 15.9%, ‘reflected upon’ in 38.6%, ‘transferred to a clinical context’ in 6.1% and ‘set in practical context’ in 16.6% of the sessions.

In Epidemiology, 72.9% sessions taught risk communication without mentioning the relevant 19 values. It was taught on the lowest teaching level ‘mentioned’ in 13.5%, ‘reflected upon’ in 3%, ‘transferred to a clinical context’ in 6% and ‘set in practical context’ on average in 4.5% of the analyzed sessions.

In Medical Psychology, risk communication was discussed on a higher scale in 61.2% of the sessions while in Epidemiology, 13.5% of the sessions taught risk commumnication on a higher intensity scale.

**Figure 3.**
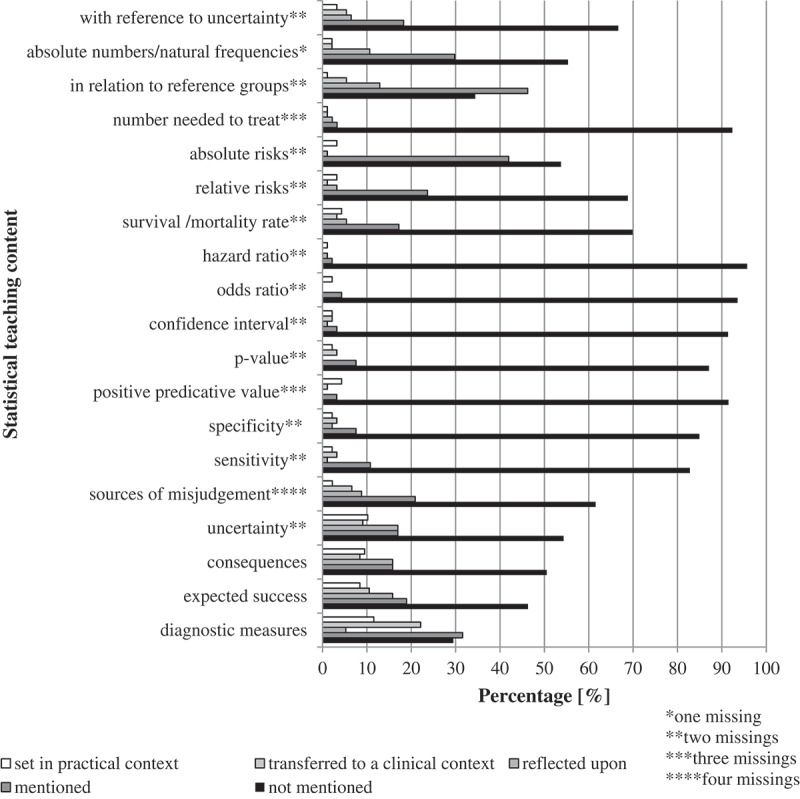
Depth of teaching content on risk communication as per defined categories of information

Figure 3 shows the overall use of statistical values in the 95 sessions where risk communication was taught.

Altogether 30,105 minutes of teaching sessions were evaluated. Of them 1,984 minutes were dedicated to communicating risks. Most departments spent between 1% and 7% time on risk communication teaching with the lowest time spent in Psychiatry, Psychosomatic Medicine and Child and Adolescent Psychiatry, and Human Genetics. Epidemiology dedicated 21.7% of its teaching time to risk communication followed by Medical Psychology with 13.7%.

### Demonstration material

Teachers used demonstration material to teach risk communication in 48 of 95 sessions. Mostly handouts (n = 22) were used while PowerPoint presentations were utilized 12 times and work-sheets five times. Trained actors as standardized patients were utilized three times. The teachers also used real patients, case studies, a TED-system and an immunization booklet twice each. A flipchart, video, examination booklet, a computer tomography scan image, diagnosis tools (picture board), sonography and a newspaper article were used once each. In three of 48 sessions, the observers did not describe the type of demonstration material provided.

**Figure 4.**
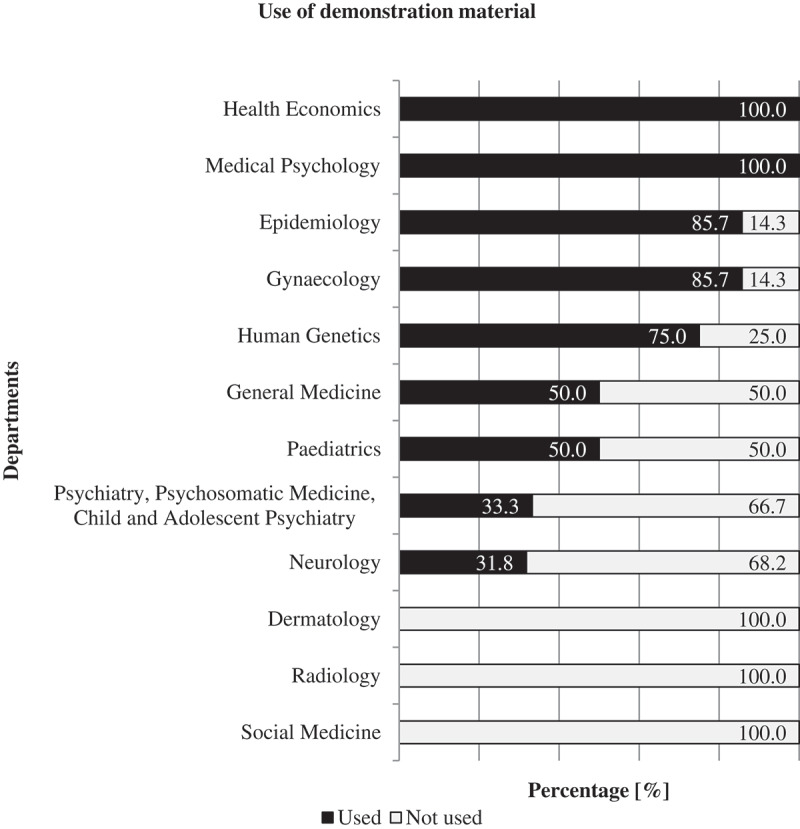
Use of demonstration material for teaching risk communication at all departments

Demonstration material was used 26 times during seminars, 13 times in lectures, four times during Practical Training, three times in SP-Training sessions and once each in BST and PBL sessions. In 43 of the 48 sessions, where demonstration material was used, the observers rated it helpful for reasons of deepening understanding (13x), learning assistance (8x), practical context (8x), additional information (8x) and interactive training (2x). Figure 4 shows the prevalence of demonstration material used in different departments.

In 55.8% of the 95 teaching sessions with risk communication, lecturers used clinical references to teach students about communicating risks. Use of clinical context was most prevalent in General Medicine (100%) and Human Genetics (87.5%) departments whereas lecturers at Social Medicine and Radiology departments did not make any clinical references. In other departments, the use of clinical context ranged between 10.5% (Dermatology) and 75% (Pediatrics). The frequency of clinical references in other departments was Gynecology 71.4%, Neurology 59.1%, Psychiatry, Psychosomatic Medicine, Child and Adolescent Psychiatry 33.3%, Medical Psychology 69.2%, Health Economics 66.7% and Epidemiology 42.9%.

Altogether 18 questions were asked in teaching sessions where risk communication was taught at all departments. Three questions each were asked in Medical Psychology, Epidemiology, Neurology and General Medicine while two questions were asked in Gynecology. No questions were asked by students at Radiology, Health Economics and psychosocial medicine departments. Seven questions were asked during lectures while five were asked during seminars. Three questions were asked in Practical Training sessions, two during BST and only once in SP-Training sessions.

In six cases, students asked about the clinical relevance of the risks taught or a clinical question e.g., about instructions for the usage of risk quantification in practical routine. In five teaching sessions, students asked about details of risk calculation. In two sessions, the questions related to communicating about risks in particular such as ‘which words should be avoided or how to be careful when talking about risks’. In three teaching sessions, the questions derived from a group discussion or were about study results. In two sessions, the observers did not reveal the questions asked.

In 60 of 95 (63.2%) teaching sessions, the observers submitted descriptive comments. Sessions were rated positively when students were taught practically relevant context (n = 13) or for impressive teaching skills (n = 5). In 31 questionnaires, observers recommended suggestions for improving the teaching sessions. In four sessions, students asked to see more practical relevance. Other suggestions included interactive teaching styles, more interaction between lecturers and students, providing primary literature/handouts, adequately communicating emotionally challenging information to patients and providing more examples for proper risk calculation.

## Discussion

This observatory survey of a curriculum provides an overview of the quality of risk communication teaching at a prestigious medical school renowned for competency-based training of future physicians. By triangulating data on prevalence, content, intensity and student-teacher interactions, we investigated whether or not medical students are taught adequately about a topic which has in recent years gained increasing importance. Our results showed that the lecturers did not devote much time to teaching students about risk communication, since the prevalence of such topics was observed in about one-fourth of the teaching sessions. The problem might, however, run deeper because even when risk communication was taught, in 90% of these teaching sessions 16 out of 19 common statistical values necessary for the interpretation of clinical data such as relative risks and absolute risks were only mentioned without further elaboration.

A descriptive analysis based on our five principal observations was used to determine which department gave the most importance to teaching risk communication. Looking at the duration of teaching by minutes alone, the departments of Epidemiology, Medical Psychology and Pediatrics taught the most about risk communication during the observation period. Although Dermatology witnessed the highest prevalence of risk communication topics, the depth of teaching when seen in combination with other variables of in-depth teaching and time spent on relevant topics was on a lower teaching scale than Epidemiology. Teaching about risk communication at Medical Psychology and Epidemiology when analyzed in combination with the teaching depth and the time spent was the most intensive. This can be understood in the context of epidemiologists as teachers, who have been trained to use statistics extensively during their medical education [31]. However, they do not have the same level of experience in setting these statistical values into practical or clinical context. Similarly, psychologists are also trained intensively on communication aspects and statistics during their college years and can transfer these skills into practical contexts [32].

Lower prevalence in Psychiatry, Psychosomatic Medicine and Child and Adolescent Psychiatry (7.1%), where students are generally taught by specialists in these fields, supports previous studies on ‘statistical illiteracy’ of medical professionals [25,33,34]. In these psychosocial medicine departments, where communication skills in general are considered a core competence and communication courses are relatively more prevalent [29], teaching on risk communication was reported on a lower qualitative scale. Psychiatrists and psychosomatic specialists may be trained on communicative and psychological aspects much more than epidemiologists but the courses in psychosocial departments were found lacking on risk communication and biostatistics teaching. This also highlights the importance of inter-professional education and training, whereby joint teaching sessions by experts on statistics and experts in communication would be helpful not only for students but also for the lecturers.

Although clinical references and demonstration material were utilized to teach students about communicating risks in almost half of the teaching sessions, comments of student observers suggested a strong desire on learning through practical context. The observers rated demonstration material as useful when employed in teaching risk communication. However, it was noted that traditional literature handouts were the most preferred choice of supplementary material while innovative methods such as TED-systems were underutilized. Instead of handouts, practical teaching aids such as simulated patients can be extremely helpful in teaching about a hands-on topic like risk communication by making it interesting for students as well as providing them with some clinical context. Nevertheless, given the positive effects of literature handouts on medical students [35], the use of any demonstration material is helpful and must be encouraged.

The topics of risk communication used to teach students though diverse did not stress enough upon the importance of primary prevention. The majority of topics (45%) used by lecturers came under the category of diseases and their associated risks while preventative measures comprised just 17% of all risk communication topics followed by consultation (16.1%), treatment (11.5%) and theoretical statistics (10.3%). Among prevention, about 20% of the topics were allocated to primary prevention while the remaining 80% focused on secondary prevention such as screening tests. Teaching on topics about primary prevention, however, could be more beneficial for future doctors as primary prevention measures bear a larger and cost-effective impact within public health [36–39]. Surprisingly, pregnancy-related themes such as prenatal diagnostics were used as risk communication topics as often as cancer although they are ethically debatable, usually not paid by insurance companies, relevant only for pregnant women/their relatives and detect only 2% of genetically transmitted diseases; whereas cancer-related health problems carry a much heavier burden [40,41].

The low number of questions during the teaching sessions on risk communication and the observers’ recommendations for interactive sessions suggested the lecturers might have failed to evoke the interest of students to a level where they wanted to participate in discussions. Some observers also suggested training lecturers on didactical skills on the failure to induce discussions during the teaching sessions. Workshops on imparting didactical skills to lecturers might be beneficial to train them on providing a more discussion-friendly environment for better knowledge transfer.

The absence of risk communication teaching in the medical curriculum has long-term effects given the fact that medical graduates quite often become medical teachers while practicing at the university hospital. When students do not get to learn crucial aspects of risk communication during school years, they are either unaware of the importance of such topics or are unable to teach them adequately later on. Although our results suggested a lack of risk communication teaching, this might be subjected to individual schools as well as teachers considering our data came from one major institution. The data obtained could not be generalized for entire curriculum based on the fact that teaching styles of individual lecturers vary from semester to semester. Also, the study was based on the observations of medical students enrolled for the same degree program and individual bias or errors cannot be ruled out. The study design was based on the aim of learning about the student perspective thus the observations must be subject to individual interpretation. Some of the questionnaires were returned with blank checkboxes on depth/intensity of teaching on specific statistical values. They have been reported as (*) missing for each data category.

## Conclusion

At a time when shared decision-making and informed consent are crucial aspects of patient-oriented health care, the importance of teaching risk communication at medical schools cannot be stressed more. Our study showed that risk communication teaching constituted a minor part of the medical curriculum with inadequate focus on important biostatistical values or practical aspects. Teaching risk communication with the aid of clinical contexts and demonstration material would not only result in an interactive educational discourse but also promote a deeper understanding of the topics. Keeping in mind the problems of miscommunication and lawsuits arising out of the ‘statistical illiteracy’ of medical professionals, our results reinforce the call for larger representation and detailed teaching of risk communication at medical schools to train students for appropriately communicating risks to patients. There lies the problem of teaching the teachers for improving their own didactical and statistical skills for better knowledge transfer, but workshops and courses can prove useful for the purpose. We recommend inter-professional teaching programs such as an epidemiologist/psychiatrist tandem that can prove extremely useful by combining the expertise of epidemiologists in biostatistics and the experience of clinicians in transferring different risk communication topics to a clinical context.

## Declarations

**Ethics approval and consent to participate**: Ethics approval was not required by the University of Heidelberg guidelines because no patients were included. The raters signed a voluntary participation contract for the study.

**Availability of data and material**: No copyrights were used in manuscript. The dataset generated and analyzed during the current study cannot be made publicly available because of the administrative and privacy concerns of the University of Heidelberg’s medical faculty and its ongoing research about prospective modernization of its medical curriculum. However, the data may be made available by the corresponding author on reasonable request.

## Appendix

**RiskComm43” ***

Dear medical students

Thank you for agreeing to participate in the survey.

Doctors have to be able to assess information about uncertainties and risks correctly and communicate them to patients in a comprehensible manner (risk communication) to enable patients to make an informed decision. Medical students in order to deal with patients should learn to take into account results from scientific work as well as the patient’s ideas in clinical decisions. Students need to learn to be able to deliberately plan and use their conversational skills, their language style and their communication techniques. To examine the extent to which teaching content on risk communication and communication competence is taught in the medical curriculum, **we ask you to consider to what extent the following statements apply. From your point of view, please mark what fits best and write your answers in the given free text fields.**

**Demographic data**

**Table:**

Name of observer:	Date: _ _. _ _. _ _ _ _
Title of course:	
Teaching format: □ Lecture □ Practical course □ Seminar □ Skills-Lab □ Job shadow □ Ward rounds training □ Tutorial □ Examination course □ POL □ Medi-KIT □ Bed-Side Teaching □ Other format: __________________________	
Name of main lecturer:	
Sex of main lecturer: □ Female □ Male	
Qualification of main lecturer: □ Student □ Lecturer	
Co-Lecturer: □ No □ Yes If yes, his/her qualification: □ Student □ Lecturer	
Maximum number of students during the course:	

For identification please repeat the course title: ____________________________________________

***How is the subject risk communication taught?***

**Table:**

Is **risk communication** taught? □ No □ Yes If yes, which **topics** are mentioned to convey the subject? (e.g., cardiovascular risk, vaccination advice, medication, genetic counselling, colorectal cancer screening,) Please fill in this sheet **separately for each topic!**	
How is the topic taught?	
How many minutes are dedicated to the topic? ___ minutes	Is any reference made to clinical cases? □ No □Yes
Is supplementary demonstration material used? □ No □ Yes If yes, which ones? (e.g. handout): _____________________________________________________________ Is the supplementary demonstration material helpful? □ No □ Yes If yes, why?	
Are there any questions on the topic from students attending the course? □ No □ Yes If yes, what?	

***What is taught about the subject risk communication?***

**Table:**

To what extent are the following objectives learned in the course?			**Not mentioned**	**Named**	**Reflected upon**	**Transferred to a clinical context**	**Set in practical context**
Diagnostic and therapeutic measures with their advantages and disadvantages			□	□	□	□	□
Expected success as well as benefits, risks and costs			□	□	□	□	□
Positive and negative consequences of waiving diagnostic and therapeutic measures			□	□	□	□	□
Uncertainty as an integral part of judgement formation and decision-making, implicit and explicit evaluation models of health and disease			□	□	□	□	□
Sources of misjudgement			□	□	□	□	□
Characteristic factors of the following diagnostic tests		- Specificity - Sensitivity - Positive predictive value - p-value - Confidence interval - Odds Ratio - Hazard Ratio - Survival/Mortality rates - Relative risks - Absolute risks - Number needed to treat	□ □ □ □ □ □ □ □ □ □ □	□ □ □ □ □ □ □ □ □ □ □	□ □ □ □ □ □ □ □ □ □ □	□ □ □ □ □ □ □ □ □ □ □	□ □ □ □ □ □ □ □ □ □ □
Communication of risks	- in relation to the reference group - in absolute numbers/natural frequencies - with the indication of uncertainty		□ □ □	□ □ □	□ □ □	□ □ □	□ □ □

Regarding the teaching of this topic, I was particularly pleased with:
What could be done to improve teaching on this topic?
Further comments:

****The RiskComm43 questionnaire can be used for research purposes provided that proper accreditation is provided to the original paper and authors.***
